# Gait comparison of unicompartmental knee arthroplasty and total knee arthroplasty during level walking

**DOI:** 10.1371/journal.pone.0203310

**Published:** 2018-08-30

**Authors:** Kyung-Wook Nha, Oog-Jin Shon, Byung-Sic Kong, Young-Soo Shin

**Affiliations:** 1 Department of Orthopaedic Surgery, Inje University, Ilsan Paik Hospital, Goyang, Korea; 2 Department of Orthopedic Surgery, College of Medicine, Yeungnam University, Daegu, Korea; 3 Department of Orthopedic Surgery, Masan Medical Center, Masan, Korea; 4 Department of Orthopedic Surgery, Veterans Health Service Medical Center, Seoul, Korea; Consorci Parc de Salut MAR de Barcelona, SPAIN

## Abstract

This meta-analysis compared the gait patterns of unicompartmental knee arthroplasty (UKA) patients and total knee arthroplasty (TKA) patients during level walking by evaluating the kinetics, kinematics, and spatiotemporal parameters. Studies were included in the meta-analysis if they assessed the vertical ground reaction force (GRF), joint moment at stance, flexion at initial contact, flexion at swing, overall range of motion (ROM), coronal knee angle at stance, walking speed, cadence, and stride length in UKA patients or TKA patients. Seven non-randomized studies met the criteria for inclusion in this meta-analysis. UKA patients and TKA patients were similar in terms of vertical GRF (95% CI: -0.36 to 0.20; P = 0.60), joint moment (95% CI: -0.55 to 0.63; P = 0.90), kinematic outcomes (95% CI: -0.72 to 1.02; P = 0.74), walking speed (95% CI: -0.27 to 0.81; P = 0.32), and cadence (95% CI: -0.14 to 0.68; P = 0.20). In contrast, the stride length (95% CI: 0.01 to 0.80; P = 0.04) differed significantly between groups. Subgroup analyses revealed that the pooled data were similar between the groups: 1st maximum (heel strike), -0.18 BW (P = 0.53); 1st minimum (mid-stance), -0.43 BW (P = 0.08); and 2nd maximum (toe off), -0.03 BW (P = 0.87). On gait analysis, there were no significant differences in vertical GRF, joint moment at stance, overall kinematics, walking speed, or cadence between UKA patients and TKA patients during level walking. However, the TKA group had significantly shorter stride length than UKA patients. Although the comparison was inconclusive in determining which types of knee arthroplasty offered the closest approximation to normal gait, we consider it important to provide better rehabilitation programs to reduce the abnormal stride length in TKA patients compared to UKA patients.

## Introduction

Total knee arthroplasty (TKA) has long been considered the gold standard operative treatment for knee arthrosis due to substantial improvements in the quality of life for patients with end stage knee osteoarthritis.[1,2] Advocates of unicompartmental knee arthroplasty (UKA) focus on the preservation of both cruciate ligaments and the remaining two knee compartments, which offers several advantages over TKA, including reduced blood loss, better range of motion, and faster recovery.[3–5] Nevertheless, TKA is more frequently performed due to the perception that TKA is a more durable repair and other influencing factors, including surgeon preference, clinic standards, and surgeon experience.[6,7] Patients who have undergone knee arthroplasty not always satisfied with the results and complained about discomfort that were not explained by traditional scoring systems, even with apparent success of arthroplasty.[8] Among several parameters to measure functional outcomes after knee arthroplasty, gait analysis was important parameter objectively. However, small number of studies about gait analysis after knee arthroplasty did not support it to have more statistical power and precision as a parameter for functional assessment.

Although many studies have evaluated gait patterns after TKA in comparison to healthy controls,[9,10] few comparative studies between UKA and TKA have been performed. Our previously published work comparing gait patterns between UKA patients and healthy controls found that results of UKA were inferior in contrary to expectations that UKA preserving cruciate ligaments would restore normal gait patterns.[9] Similarly, gait patterns of UKA were expected to be closer to normal gaits than TKA which is sacrificing cruciate ligaments and we wanted to evaluate this question of whether UKA is able to restore more physiological gait than TKA. Additionally, we attempted to include randomized and non-randomized studies because non-randomized studies can complement or address some randomized controlled studies limitations, such as short follow-up periods, small sample size, highly selected populations, high cost, and ethical restrictions.[11]

Therefore, this meta-analysis was designed to compare gait patterns of UKA and TKA patients using all recorded gait parameters. It was hypothesized that TKA patients with an ACL sacrificing procedure would have an impaired the gait pattern compared to UKA patients.

## Materials and methods

This meta-analysis was conducted according to the guidelines of the preferred reporting items for systematic reviews and meta-analysis (PRISMA) statement (S1 PRISMA Checklist)

### Data and literature sources

Multiple comprehensive databases, including MEDLINE (January 1, 1976 to December 31, 2017), EMBASE (January 1, 1985 to December 31, 2017), Web of Science (January 1, 1980 to December 31, 2017), SCOPUS (January 1, 1980 to December 31, 2017), and the Cochrane Library (January 1, 1987 to December 31, 2017) were searched for studies that compared the gait patterns of UKA and TKA patients, as assessed with knee kinetics, kinematics or spatiotemporal data. There were no restrictions on language. Search terms used in the title, abstract, MeSH, and keywords fields included (‘gait’ [MeSH] OR ‘walking’ [MeSH] OR ‘knee joint’ [MeSH] OR ‘osteoarthritis, knee’ [MeSH] OR ‘spatiotemporal’ [MeSH]) AND ‘gait’ [tiab] OR ‘unicompartmental knee arthroplasty’ [tiab] OR ‘total knee arthroplasty’ [tiab] OR ‘walking speed’ [tiab] OR ‘stride length’ [tiab] OR ‘cadence’ [tiab] OR ‘kinetics’ [tiab] OR ‘ground reaction forces’ [tiab] OR ‘joint moments’ [tiab] OR ‘kinematics’ [tiab]). After the initial electronic search, relevant articles and their bibliographies were manually searched.

### Study selection

From the title and abstract, two reviewers independently selected relevant studies for full review. The full text of an article was reviewed if the abstract did not provide sufficient data for a decision. Studies were included in the meta-analysis if they (1) evaluated human knees previously undergone UKA or TKA for osteoarthritis; (2) had an evidence level of 1 to 3; (3) reported retrospective or prospective comparisons of gait analysis between UKA and TKA patients; (4) included data of at least one of the following three parameters of knee joint: kinetic and/or kinematic characteristics in the coronal or sagittal plane while walking with collected spatiotemporal parameters. To obtain kinetic and spatiotemporal data, patients walked at their natural or maximum pace on a long force platform or treadmill until consistent velocity was gained. Vertical ground reaction force (GRF) and joint moments were normalized to body weight (BW), and maximum values were calculated. Joint moments consisted of peak varus moment and peak extension moment. Spatiotemporal parameters included walking speed, stride length, and cadence. Knee flexions at initial contact, at swing phase, overall sagittal ROM at stance phase, and coronal knee angle at stance phase were analyzed as kinematic data. Knee angles were calculated only in the sagittal and coronal planes because insufficient detail in reporting prevented valid calculation of the effective size; (5) fully reported the number of subjects in each group (UKA and TKA), and the means and standard deviations for the three parameters; and (6) used adequate statistical methods to compare these parameters between groups. Studies were excluded if they (1) constantly need walking aids because the patients are considered not adequately rehabilitated; (2) had prior joint arthroplasties in any joint of the lower limbs; (3) had missing or inadequate outcome data, such as standard deviations or ranges of values; or (4) were case series, expert opinions, reviews, commentaries, or editorials.

### Data extraction

Two reviewers independently recorded data from each study using a predefined data extraction form and resolved any differences by discussion. Recorded variables of gait analysis for UKA and TKA patients were included. Sample size and the means and standard deviations of kinetic, kinematic, and spatiotemporal parameters in each group were also recorded. If these variables were not included in the articles, the standardized mean difference was calculated from the p-value and sample size.

### Methodological quality assessment

Two reviewers independently assessed the methodological quality of the studies. For the Newcastle-Ottawa Scale, as recommended by the Cochrane Non-Randomized Studies Methods Working Group, we assessed studies based on three criteria: selection of the study groups, comparability of the groups, and ascertainment of either the exposure or the outcome of interest for case-control and cohort studies. Studies of high quality were defined as those with scores higher than 6 points. Two reviewers resolved all differences by discussion, and their decisions were subsequently checked by a third investigator. Publication bias could not be assessed in these trials. Tests for funnel plot asymmetry are typically performed only when at least ten studies are included in the meta-analysis.[12] As our analysis included only seven studies, tests for asymmetry were not performed because these tests would not be able to differentiate asymmetry from chance.

### Data synthesis and statistical analysis

The main outcomes of the meta-analysis were the standardized mean differences (SMDs) in kinetic and spatiotemporal parameters between UKA and TKA patients because these outcome measurements among studies were made on the different ways; and the weighted mean difference (WMD) in kinematic parameters between these patients. For all comparisons, SMDs or WMDs and 95% confidence intervals (CIs) were calculated for continuous outcomes. Heterogeneity was determined by estimating the proportion of between-study inconsistencies due to actual differences between studies, rather than differences due to random error or chance. The I^2^ statistic was used with values of 25%, 50%, and 75% considered as low, moderate, and high heterogeneity, respectively. The random effect model was used to calculate the pooled effect size because we determined that the populations and interventions of the studies integrated in the current meta-analysis were heterogeneous. All statistical analyses were performed with the RevMan version 5.3 software and Stata version 14.2 static software. In order to explore a potential source of heterogeneity, subgroup analysis based on the classical division of gait at stance phase was performed for vertical GRF. As a result, three subgroups were created for each of the two study groups: 1st maximum (heel strike), 1st minimum (mid-stance), and 2nd maximum (toe off). Additional subgroup analysis based on study type was performed for the 1st maximum (heel strike). As a result, two subgroups were created for each of the two study groups: prospective comparative studies and retrospective comparative studies. Sensitivity analysis was performed by excluding one eligible study at a time. One study [13] with results including top walking speed was included because walking speed is probably the cause for limited between-group differences in gait analysis and another study [14] with follow-up less than 6 months were included. The time period was chosen to reduce the effects of surgery and healing on gait. Pooling of data was feasible for four outcomes of interest: vertical GRF, overall joint moment, overall kinematic results, and overall spatiotemporal results.

## Results

### Study identification

**Fig 1:**
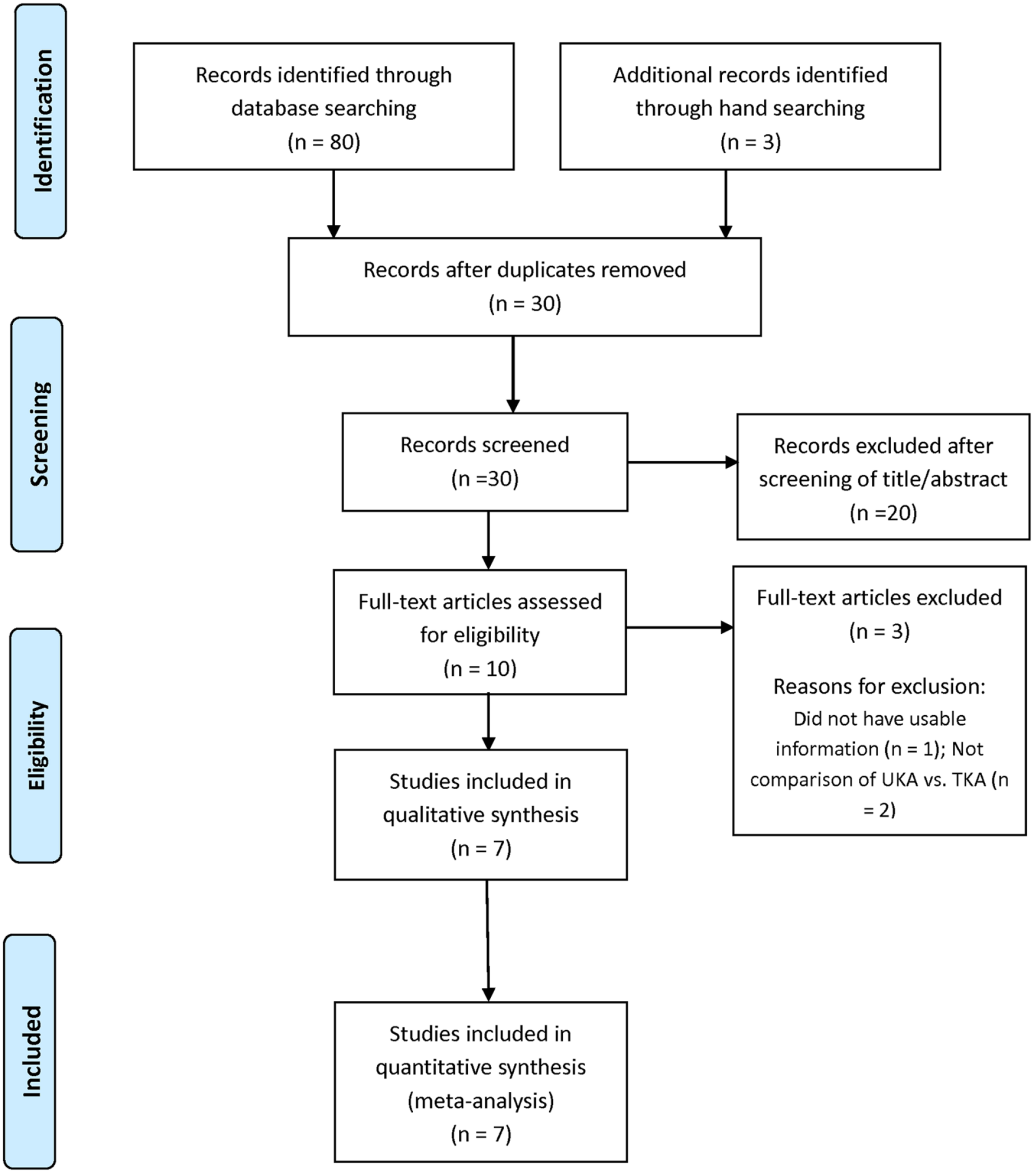
Preferred reporting items for systemic reviews and meta-analyses (PRISMA)flow diagram of literature selection.

Details on study identification, inclusion, and exclusion are summarized in Fig 1. An electronic search yielded 20 studies in PubMed (MEDLINE), 23 in EMBASE, 16 in Web of Science, 18 in SCOPUS, and 3 in the Cochrane Library. Three additional publications were identified through manual searching. After removing 53 duplicates, 30 studies remained. Of these, 20 were excluded based on abstract and full-text article review, and an additional three studies were excluded because they had unusable information or made inappropriate group comparisons. This eventually resulted in seven studies that were included in the meta-analysis.[13–19]

### Study characteristics, patient populations, and quality assessment of the included studies

The seven studies included 82 UKA patients and 99 TKA subjects who underwent gait analysis that included kinetic, kinematic, or spatiotemporal parameters. One study (1 PCS) compared prospectively measured parameters, whereas the other six studies compared parameters measured by retrospective chart review. Seven studies compared groups according to walking speed; five compared vertical GRF; four compared cadence; three compared varus moment at stance, coronal knee angle at stance, and stride length; and two compared extensor moment at stance, flexion at initial contact, flexion at swing, and overall ROM at stance. The quality of the seven studies included in the meta-analysis is summarized in Table 1. Non-RCTs (one PCS and six RCSs) were of high quality (Newcastle-Ottawa Scale > 6).

**Table 1:** Summary of patient characteristics of the included studies.

Study	Year	Study type	Mean age (years)		Sample size (M/F)		Weight(kg)/Height(m)		Prosthesis properties		Time of gait analysis from surgery (months)	Quality score	Measured parameters
			UKA	TKA	UKA	TKA	UKA	TKA	UKA	TKA			
Braito et al.[14]	2016	PCS	65.7	66.4	15(8/7)	17(6/11)	NA	NA	Oxford	Scorpio	At least 2	NOS 7	VGRF, VMS, EMS, FIC, FS, ORMS, CKAS, WS, CD, SL,
Calliess et al.[15]	2014	RCS	58.0	61.3	2(1/1)	4(2/2)	NA	NA	Triathlon	Triathlon	Mean 12	NOS 8	WS, CD
Choy et al.[16]	2007	RCS	70.3	70.3	12(0/12)	12(0/12)	NA	NA	Oxford	LCS	At least 6	NOS 7	VGRF, VMS, EMS, FIC, FS, ORMS, CKAS, WS, CD, SL,
Jones et al.[17]	2016	RCS	65.0	68.0	12(N/S)	12(N/S)	88.8/1.75	83.7/1.67	Oxford	Genesis II	At least 12	NOS 8	VGRF, WS
Komnik et al.[18]	2016	RCS	60.5	60.0	13(7/6)	11(7/4)	80.4/1.70	82.1/1.73	UHFKS	SIGMA/Genesis II	Mean 20	NOS 9	VMS, CKAS, WS,
Stacoff et al.[19]	2006	RCS	67.2	67.3	5(3/2)	20(9/11)	77.7/1.76	79.0/1.70	Allegretto	LCS/INNEX	At least 13	NOS 8	VGRF, WS
Wiik et al.[13]	2013	RCS	65.9	67.8	23(10/13)	23(9/14)	86.7/1.70	84.0/1.69	NA	NA	At least 12	NOS 8	VGRF, WS, CD, SL

Abbreviations: PCS, prospective comparative study; RCS, retrospective comparative study; M, male; F, female; UKA, unicompartmental knee arthroplasty; TKA, total knee arthroplasty; NA, not available; LCS, low-contact-stress; UHFKS, unicompartmental high flex knee system; NOS, Newcastle-Ottawa Scale; VGRF, vertical ground reaction force (BW); VMS, varus moment at stance (Nm/(kg∙m)); EMS, extensor moment at stance (Nm/(kg∙m)); FIC, flexion at initial contact (°); FS, flexion at swing (°); ORMS, overall range of motion at stance (°); CKAS, coronal knee angle at stance(°); WS, walking speed (m/s); CD, cadence (step/min); SL, stride length (cm)

### Kinetic analysis

**Fig 2:**
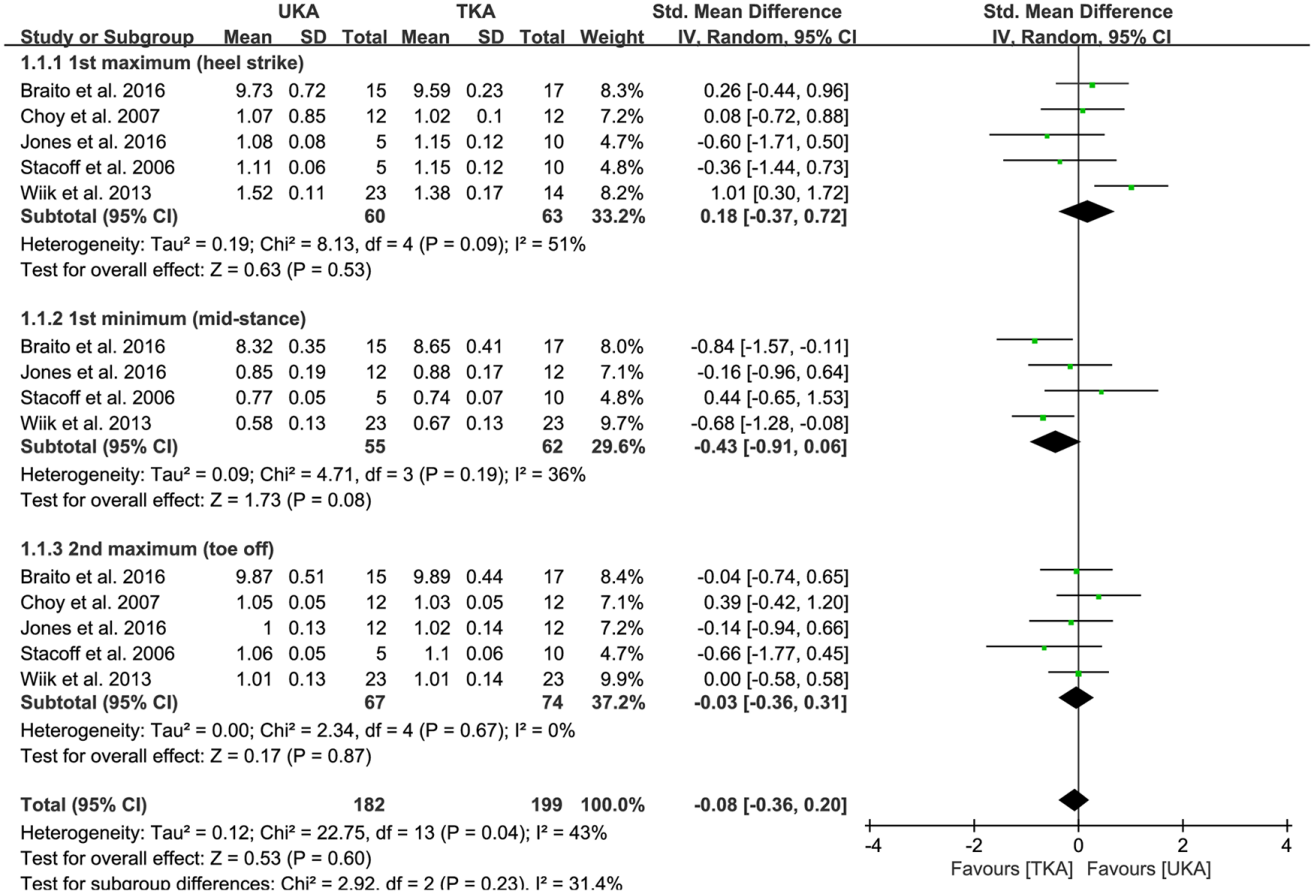
Results of aggregate analysis for comparison of vertical ground reaction force (GRF) between UKA and TKA, including subgroup analysis by 1^st^ maximum (heel strike), 1^st^ minimum (mid-stance), and 2^nd^ maximum (toe off).

**Fig 3:**
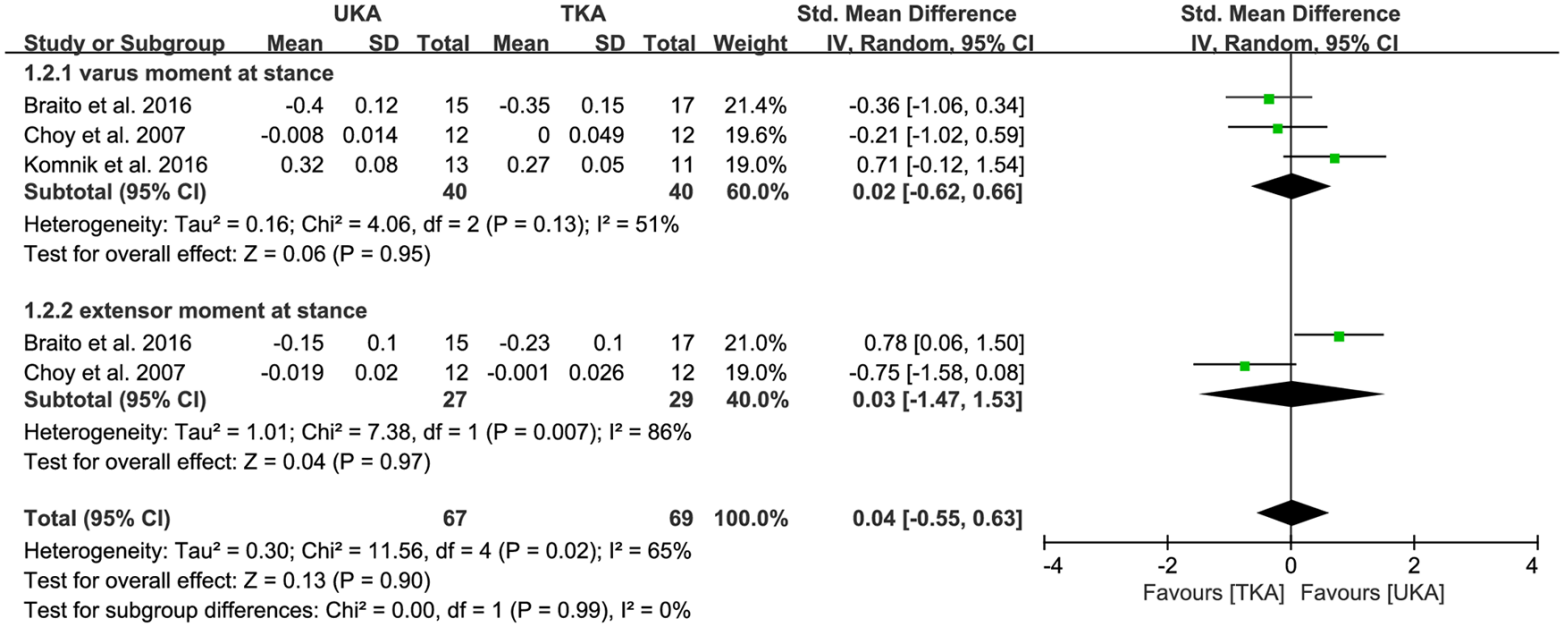
Results of aggregate analysis for comparison of joint moment in the sagittal and coronal plane between UKA and TKA.

Of the seven studies, vertical GRF between UKA (n = 182) and TKA patients (n = 199) was compared in five studies. The pooled data showed that the mean vertical GRF was -0.08 BW less for UKA patients than TKA patients, but this difference was not significant (95% CI: -0.36 to 0.20 BW; P = 0.60; I^2^ = 43%, Fig 2). Five studies were assigned to the 1st maximum (heel strike) and 2nd maximum (toe off) subgroups. Four studies were assigned to the 1st minimum (mid-stance). The 1st maximum (heel strike) subgroup was 0.18 BW greater in UKA patients than TKA patients, but this difference was not significant (95% CI: -0.37 to 0.72 BW; P = 0.53; I^2^ = 51%, Fig 2). The 1st minimum (mid-stance) subgroup showed -0.43 BW less in UKA patients than in TKA patients, but this difference was also not significant (95% CI: -0.91 to 0.06 BW; P = 0.08; I^2^ = 36%, Fig 2). Likewise, the 2nd maximum (toe off) subgroup was -0.03 BW less in UKA patients than TKA patients, but this difference was not significant (95% CI: -0.36 to 0.31 BW; P = 0.87; I^2^ = 0%, Fig 2). In addition, one study was assigned to the prospective comparative study subgroup. Four studies were assigned to the retrospective comparative study subgroup. The prospective comparative study subgroup was 0.26 BW greater in UKA patients than TKA patients, but this difference was not significant (95% CI: -0.44 to 0.96 BW; P = 0.46; I^2^ = NA, Table 2). Likewise, the retrospective comparative study subgroup was 0.11 BW greater in UKA patients than in TKA patients, but this difference was also not significant (95% CI: -0.63 to 0.86 BW; P = 0.76; I^2^ = 63%, Table 2). Of the seven studies, three compared the varus moment at stance between UKA (n = 67) and TKA patients (n = 69). In the pooled data, UKA patients had 0.02 BW greater mean varus moment at stance than TKA patients, but this difference was not significant (95% CI: -0.62 to 0.66 BW; P = 0.95; I^2^ = 51%, Fig 3). Similarly, two studies compared the extensor moment at stance between UKA (n = 27) and TKA patients (n = 29). According to the pooled data, UKA patients had 0.03 BW greater mean extensor moment at stance than TKA patients, but this difference was not significant (95% CI: -1.47 to 1.53 BW; P = 0.97; I^2^ = 86%, Fig 3). The results of the sensitivity analysis were not significantly different from those of the original analyses, indicating that the findings were robust to the decisions made in the process of obtaining them (Table 3).

**Table 2:** Summary of standardized mean difference for outcomes of subgroup analysis in terms of study type.

Outcome or subgroup	Number of studies	Participants (UKA/TKA)	ES (95% CI)	I^2^ (%)	*P* value
VGRF (heel strike)			SMD		
All	5	60/63	0.18 (-0.37 to 0.72)	51	0.53
Subgroup analysis					
PCS	1	15/17	0.26 (-0.44 to 0.96)	NA	0.46
RCS	4	45/46	0.11 (-0.63 to 0.86)	63	0.76

VGRF, vertical ground reaction force; PCS, prospective comparative study; RCS, retrospective comparative study; UKA, unicompartmental knee arthroplasty; TKA, total knee arthroplasty; ES, effect size; CI, confidence interval; SMD, standardized mean difference; NA, not available

**Table 3:** Sensitivity analysis.

Study	Parameter	Before exclusion	After exclusion	Statistical significance
Braito et al.[14] (2016)	Vertical GRF	SMD = -0.08, 95% CI = -0.36 to 0.20, Z = 0.53, P = 0.60	SMD = -0.04, 95% CI = -0.36 to 0.29, Z = 0.22, P = 0.83	No difference
	Joint moment	SMD = 0.04, 95% CI = -0.55 to 0.63, Z = 0.13, P = 0.90	SMD = -0.09, 95% CI = -0.91 to 0.74, Z = 0.20, P = 0.84	No difference
	Kinematic results	SMD = 0.15, 95% CI = -0.72 to 1.02, Z = 0.34, P = 0.74	SMD = 0.83, 95% CI = -1.18 to 2.85, Z = 0.81, P = 0.42	No difference
	Spatiotemporal results	SMD = 0.32, 95% CI = 0.05 to 0.60, Z = 2.29, P = 0.02	SMD = 0.83, 95% CI = -1.18 to 2.85, Z = 0.81, P = 0.42	Difference
Wiik et al.[13] (2013)	Vertical GRF	SMD = -0.08, 95% CI = -0.36 to 0.20, Z = 0.53, P = 0.60	SMD = 0.21, 95% CI = -0.13 to 0.56, Z = 1.20, P = 0.23	No difference
	Spatiotemporal results	SMD = 0.32, 95% CI = 0.05 to 0.60, Z = 2.29, P = 0.02	SMD = 0.20, 95% CI = -0.11 to 0.50, Z = 1.26, P = 0.21	Difference

GRF, ground reaction force; SMD, standardized mean difference; CI, confidence interval

### Kinematic analysis

**Fig 4:**
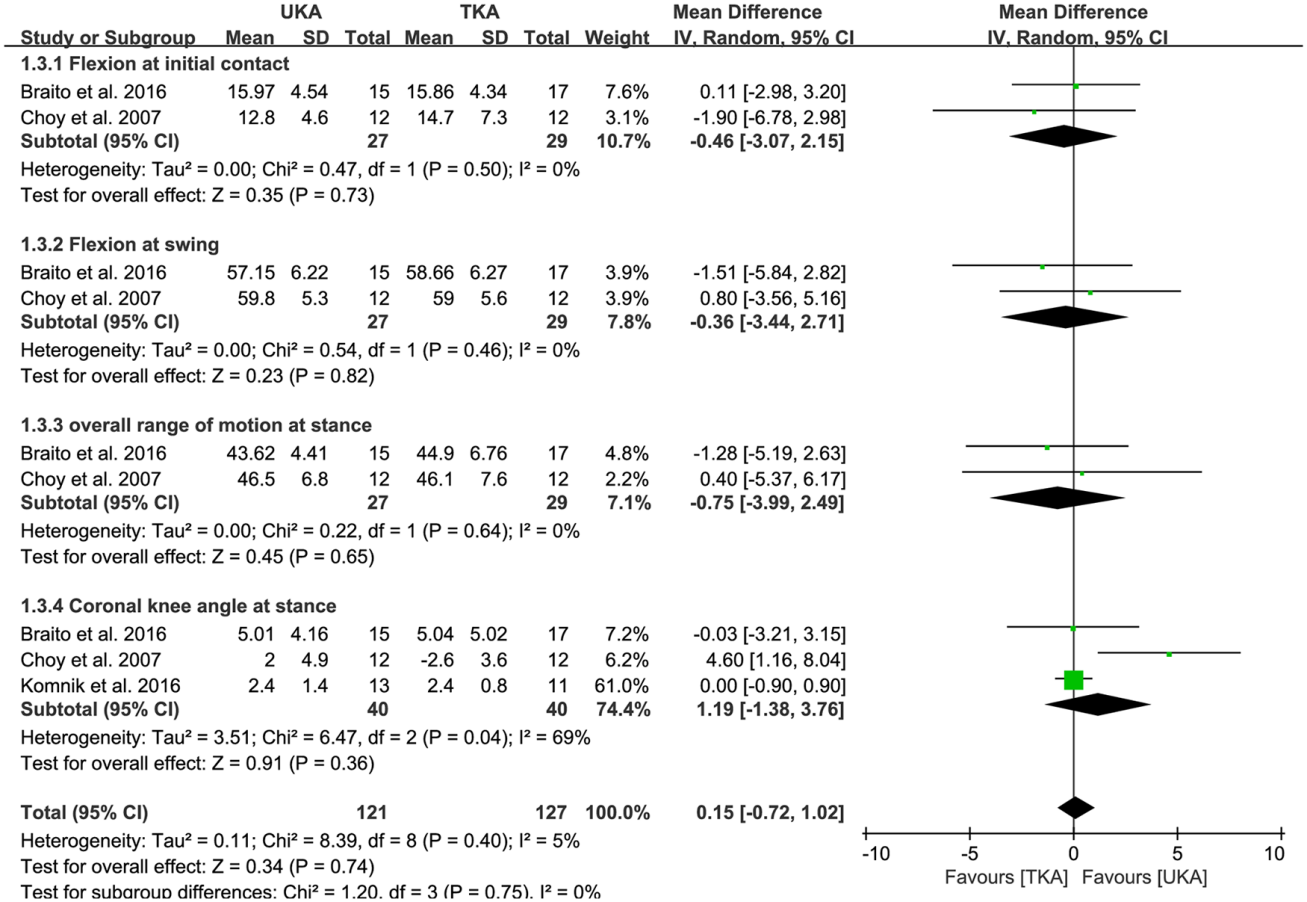
Results of aggregate analysis for comparison of overall kinematics in the sagittal and coronal plane between UKA and TKA.

Of the seven studies, flexion at initial contact between UKA (n = 27) and TKA patients (n = 29) were compared in two studies. The pooled data indicated that mean flexion at initial contact was less by -0.46° in UKA patients than TKA patients, but this difference was not significant (95% CI: -3.07 to 2.15°; P = 0.73; I^2^ = 0%, Fig 4). The pooled mean difference of flexion at swing was -0.36° less in UKA patients than TKA patients, but this difference was not significant (95% CI: -3.44 to 2.71°; P = 0.82; I^2^ = 0%, Fig 4). The pooled data showed that mean overall ROM at stance was less by -0.75° in UKA patients than TKA patients, but this difference was also not significant (95% CI: -3.99 to 2.49°; P = 0.65; I^2^ = 0%, Fig 4). Three studies compared coronal knee angle at stance between UKA (n = 40) and UKA patients (n = 40). The pooled mean difference in coronal knee angle at stance was 1.19° (95% CI: -1.38 to 3.76°; P = 0.36; I^2^ = 69%, Fig 4). The results of sensitivity analysis were not significantly different from those of the original analyses (Table 3).

### Spatiotemporal analysis

**Fig 5:**
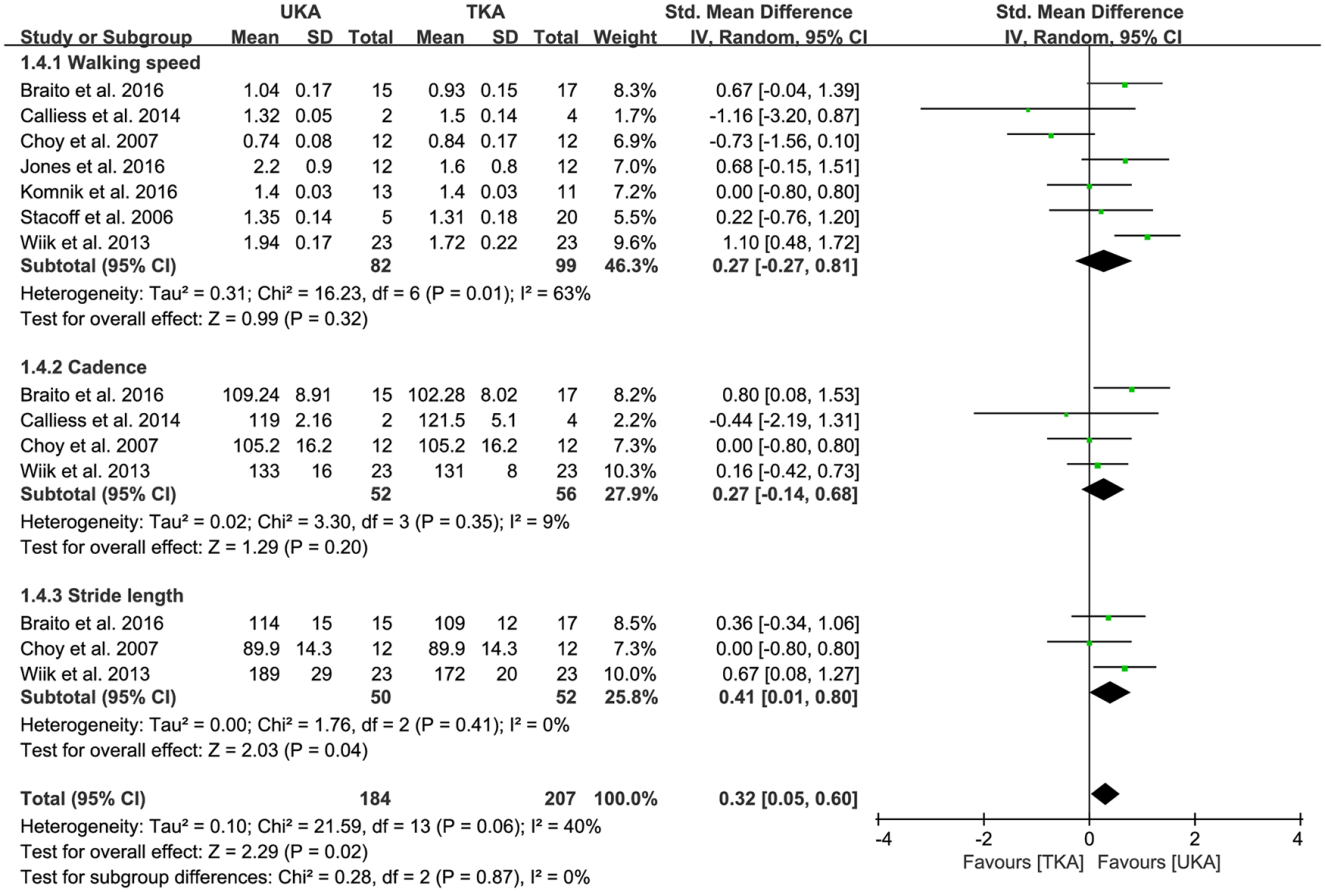
Results of aggregate analysis for comparison of spatiotemporal parameters, including walking speed, cadence, and stride length between UKA and TKA.

All seven studies compared walking speed between UKA (n = 82) and TKA patients (n = 99). The pooled data on walking speed was 0.27 m/s greater in UKA patients than TKA patients, but this difference was also not significant (95% CI: -0.27 to 0.81 m/s; P = 0.32; I^2^ = 63%, Fig 5). Likewise, four studies compared the cadence between UKA (n = 52) and TKA patients (n = 56). The pooled data on cadence was 0.27 steps/min greater in UKA patients than TKA patients, but this difference was also not significant (95% CI: -0.14 to 0.68 steps/min; P = 0.20; I^2^ = 9%, Fig 5). Conversely, three studies compared stride length between UKA (n = 50) and TKA patients (n = 52). The pooled data on stride length was 0.41 cm (95% CI: 0.01 to 0.80 cm; P = 0.04; I^2^ = 0%, Fig 5), indicating that stride length was significantly shorter in TKA patients than UKA patients. The results of sensitivity analysis were significantly different from those of the original analysis for spatiotemporal parameters (Table 3).

### Meta-regression analysis

The results of meta-regression analysis are summarized in Table 4. Sex was not significantly associated with the mean difference in stride length, indicating that sex did not affect the mean difference in stride length between UKA and TKA.

**Table 4:** Meta-regression analyses of gender and difference in mean stride length for UKAs and TKAs.

Variable	Coefficient	Standard error	P value	95% confidence interval
Stride length				
Gender	0.541	0.469	0.455	-5.422 to 6.504

## Discussion

The principal findings from this meta-analysis were that UKA and TKA patients during level walking did not have significant differences in vertical GRF, joint moment at stance, overall kinematics, walking speed, or cadence. However, the TKA group had significantly shorter stride length than the UKA group.

Walking speed is an important parameter to evaluate functional outcome after knee arthroplasty and small increases of 0.1 m/s in the walking speed may alter outcomes.[19, 20] However, there is no consensus on the walking speed of patients treated with knee arthroplasty. Generally, muscle power is not restored to healthy control levels regardless of arthroplasty procedures, suggesting reduced differences on walking speed are similar when compared to the healthy control group.[21–23] In contrast, UKA should lead to normal walking speed and increased cadence and stride length, resulting from preserving both cruciate ligaments, which play a major role in proprioceptive joint control, and substituting only the medial or lateral knee compartment.[24] The results of this meta-analysis do not support the theoretical advantage of UKA over TKA because all but one of the tested parameters, including walking speed, one of the most important parameters for assessing the functional outcomes of arthroplasty procedures, did not differ between the two groups. Although UKA led to significantly longer stride length than TKA, the difference was just marginally significant (p-value of 0.04). The less than 0.5 cm differences observed between the two groups may have little clinical relevance and likely falls within the range of measurement error. Additionally, sex would be influential on the estimated stride length during level walking even though numerous gait variables change with stride length. Our study found that sex did not have much impact on stride length in the meta-regression analysis.

Although TKA should obtain neutral alignment, the range of an adequate coronal knee alignment after UKA is still debated. Previous studies have reported a reduced risk of failure when the tibial component was inserted with close-to-neutral alignment,[25, 26] whereas postoperative slight valgus or varus alignment did not compromise the overall outcomes or final UKA survivorship.[27, 28] Our meta-analysis showed that the UKA and TKA groups did not show a significant difference in coronal knee angle at stance. It should be noted that compensatory mechanisms in the UKA group such as increased lateral trunk lean have been reported to decrease knee adduction moments during level walking, similar to the findings in knees with TKA.[9] In addition, we found that the pooled mean difference in coronal knee angle between UKA and TKA was 1.19° during the stance phase of level walking. These findings differ from previous reports that mean difference in coronal knee angle between the two methods was 6.5° during the stance phase of stair descent.[29] This indicates that discrepancies between UKA and TKA in terms of kinematics and kinetics would be more apparent during neurophysiologically more demanding locomotion tasks.

Our meta-analysis also showed that the measurement methods of the included studies were primarily optoelectronic, force plate and inertial measurement unit (IMU) based, even though most previous studies reporting normal gait mechanics in UKA patients were conducted by means of fluoroscopic analysis.[30–32] This is probably because fluoroscopic analysis is limited to relatively simple tasks that can be performed within the small measurement volume of the fluoroscope, whereas optoelectronic analysis involves a far more convenient and comprehensive assessment using force plates and is less limiting for functional task evaluation than fluoroscopic analysis.[33] In addition, IMU-based analysis demonstrates the advantages of higher flexibility, portability and adaptability, in contrast to fluoroscopic analysis for measuring knee joint angles.[34]

Previous studies have reported that knee flexion in the sagittal plane during the stance and swing phase is positively correlated with walking speed. This finding suggested that increased knee flexion at the stance phase for faster walking speed may allow an even distribution of knee forces over a wider region of tibiofemoral cartilage.[35] Interestingly, our meta-analysis showed that the mean knee flexion angle after TKA did not show a significant difference during the stance and swing phase. However, there was a trend toward TKA patients having a greater knee flexion angle than UKA patients, even with an 8% slower walking speed. Discrepancies between the two groups may result from residual flexion deformity and impaired quadriceps function, even though flexion deformity remains to some extent one year after UKA, but not as much as in TKA.[16]

This study has several limitations. All seven studies were in our inclusion of only Level II and III trials, so there was inherent heterogeneity due to uncontrolled bias, even though the studies had high quality scores. Second, the use of a wide variety of gait analysis systems and modeling techniques, as well as variability in subject characteristics and prosthetic designs were different among studies, which could have negatively affected the assessment of gait pattern. Third, although there were four studies including a healthy control group in the current meta-analysis, it could not compare TKA, UKA, and controls simultaneously due to the limitations of statistical analysis. Hence, future large-scale studies should include TKA, UKA and controls in order to investigate the potential benefits of UKA over TKA. Finally, we did not compare preoperative gait data, which may influence the postoperative gait pattern. Further studies are required to definitively investigate the gait pattern prior to operation for the two groups.

## Conclusions

On gait analysis, there were no significant differences in vertical GRF, joint moment at stance, overall kinematics, walking speed, or cadence between UKA patients and TKA patients during level walking. However, TKA patients had a significantly shorter stride length than UKA patients. Although the comparison was inconclusive in determining which types of knee arthroplasty offered the closest approximation to normal gait, we consider it important to provide better rehabilitation programs to reduce abnormal stride length in TKA patients compared with UKA patients.

## Supporting information

S1 PRISMA Checklist PRISMA checklist. (DOC)
